# A Flexible Piezoelectric Nanogenerator Based on Aligned P(VDF-TrFE) Nanofibers

**DOI:** 10.3390/mi10050302

**Published:** 2019-05-05

**Authors:** Sujian You, Lingling Zhang, Jinzheng Gui, Heng Cui, Shishang Guo

**Affiliations:** 1 Wuhan University Shenzhen Research Institute, Shenzhen 518000, China; 200731230029@whu.edu.cn (S.Y.); llzhang@whu.edu.cn (L.Z.); jinzhenggui@whu.edu.cn (J.G.); cuiheng@whu.edu.cn (H.C.); 2 College of Mathematics and Physics, Fujian University of Technology, Fuzhou 350118, China; 3 Key Laboratory of Artificial Micro- and Nano-structures of Ministry of Education, School of Physics and Technology, Wuhan University, Wuhan 430072, China

**Keywords:** P(VDF-TrFE), aligned nanofibers, piezoelectric nanogenerator

## Abstract

Aligned P(VDF-TrFE) nanofibers are successfully fabricated by advanced electrospinning. The aligned feature of the nanofibers is achieved by using parallel electrodes, which is fabricated by lithography and wet etching, and a rotating drum collector. Scanning electron microscope (SEM) images show that the nanofibers are highly ordered with a smooth surface and uniform diameter. X-ray diffraction (XRD) and Fourier Transform Infrared spectrum (FTIR) tests indicate that the fibers contain high β phase content. The nanogenerator based on aligned P(VDF-TrFE) nanofibers exhibits good electric performance with a maximum output voltage as high as 12 V and peak-peak short circuit current about 150 nA, highlighting the potential application of P(VDF-TrFE) on self-powered and wearable devices.

## 1. Introduction

Scavenging waste energy from human movement or ambient environments has become an interesting topic recently [1,2,3,4,5,6]. To date, a wide spectrum of mechanical energy scavenging techniques has been successfully demonstrated, including tribo-electricity [2,7] and piezoelectricity [7,8,9,10]. For example, the triboelectric nanogenerators (TENGs) have shown extremely high energy conversion efficiency and high output voltage [11]. Various designs and approaches have been reported for harvesting energy using TENG [12]. However, their relatively large size makes them unfavorable for use in small-size, and also requires sophisticated device structures to ensure enough resilience for the charge separation, otherwise there is no output current [13]. Furthermore, the use of polymers as electrification materials in TENGs causes wear and tear on two contact surfaces, which has led to doubts regarding their long-term stability [14]. Meanwhile, generators based on piezoelectricity have become the most widely used devices in various applications due to their high sensitivity, stability, fast-response, and parallel-reading detections of spatial pressure distributions [15,16,17,18]. After ZnO nanowire arrays were demonstrated [19], several materials with different architectures for piezoelectric generators have been reported [15,16,20,21]. Although the inorganic materials such as Lead Zirconate Titanate (PZT) ceramic or BaTO_3_ (BTO) have high piezoelectric coefficients, the fragility of them limits their integrations in flexible electronic devices, and also makes them easy to be damaged during working [22,23]. Polyvinylidene fluoride (PVDF) and its copolymer P(VDF-TrFE) are emerging piezoelectric polymer with unique features including biocompatibility, high power density, and high flexibility, especially promising for wearable devices powered by such flexible sensors [24] and harvesters [14,25,26]. Recently, various PVDF-based generators with different functional morphologies, including multiple structure films [27,28,29], nanofibers [30], nanotubes [31], and other nano-patterns [32] have been used for the design of novel flexible wearable/implantable devices. In particular, previous reports described that the electric field and associated extensional forces produced by electrospinning methods naturally cause local poling for piezoelectric nanofibers [20,33,34]. Fang et al. [33] demonstrated a one-step fabrication of piezoelectric PVDF nanofibers without any extra poling treatment that can be used to convert mechanical energy. Persano et al. [34] indicate that aligned P(VDF-TrFE) nanofibers can be formed into flexible, free-standing sheets, by use of electrospinning onto a fast rotating collector. Thereby enabling excellent response and high piezoactive β-fraction without further processing. And the high volumetric densities of aligned nanofibers have more potential in actual device fabrication. At present, those aligned nanofibers are mainly obtained by means of gap collectors [35] or rotating collectors [34]. But gap collectors generally have disadvantages in producing large area, and rotating collectors need high speed to obtain uniform structures, making it unsuitable for realizing large area and multilayered aligned arrays. In this article, we demonstrate a novel method combining gap collectors and rotating collectors to obtain a large area of aligned P(VDF-TrFE) nanofibers. The unique parallel electrodes can also be adopted to improve the device performance. And then, a simple P(VDF-TrFE) nanofibers-based piezoelectric nanogenerator (PENG) is fabricated. The PENG exhibits a high output voltage about 12 V, and peak-peak short-circuit current about 150 nA. These findings will promote the development in self-powered devices and wearable energy harvesters.

## 2. Experimental Procedure

**Figure 1:**
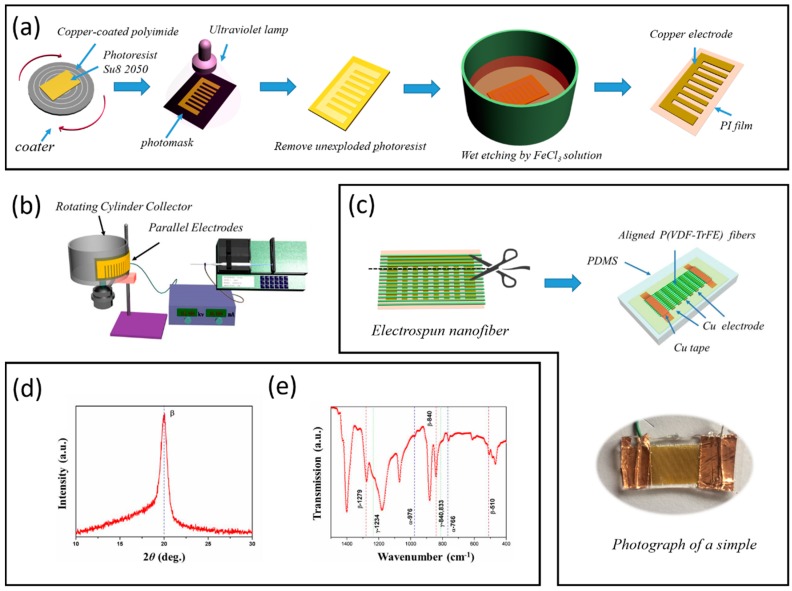
(**a**) Schematic diagram of flexible parallel copper electrode fabrication, (**b**) the schematic of electrospinning progress, (**c**) nanogenerator fabrication, (**d**) X-ray diffraction (XRD) pattern of the P(VDF-TrFE) nanofibers, (**e**) infrared spectra of the P(VDF-TrFE) nanofibers.

The preparation of P(VDF-TrFE) nanofibers. A P(VDF-TrFE) solution was prepared by adding 1 g P(VDF-TrFE) (70/30) powder to 5 mL of dimethyl formamide/acetone (7/3 v/v), and then stirring until homogeneous at room temperature. The electrospinning procedure was shown in Figure 1b, the voltage applied on the nozzle was about 15 kV, and a cylindrical collector (diameter = 18 cm) was placed at a distance of about 15 cm. A unique feature of this setup is a flexible parallel copper electrode which is fabricated by a standard lithography process and wet etching method (Figure 1a). The electrode was pasted on the side of the cylindrical collector that is used as the collecting device, the speed of the cylindrical collector was about 1000 RPM. The as-prepared P(VDF-TrFE) nanofibers are approximately 1 cm in width and 2 cm in length. The thickness has a range of 5–10 μm, which depends on the size of the electrode and spinning time. The electrospun membranes were still placed in the high-speed collector with room temperature for 5 h before desiccation.

Nanogenerator fabrication. As shown in Figure 1c, after electrospinning, the joints of the parallel electrodes are cut off by a scissor. The PENG was fabricated simply by establishing electrical contacts to two sides polymer [4]. 

Device characterization. The crystalline structures of nanofibers were characterized by X-ray diffraction (XRD, Bruker AXS, D8-ADVANCE, Karlsruhe, Germany). Room temperature Fourier Transform Infrared spectrum (FTIR) spectra were recorded on NICOLET 5700 FTIR spectrometer at 4 cm^−1^ resolution in the range of 1500–400 cm^−1^. The morphologies of electrospun P(VDF-TrFE) nanofibers were observed using a scanning electron microscope (SEM, Hitachi S-4800, Tokyo, Japan). Each sample was sputter-coated with gold for analysis. The average for diameter was calculated using appropriate software and on the basis of SEM images.

## 3. Results and Discussion

As known, PVDF is a semi-crystalline polymer with a complex structure, and it presents five distinct crystalline phases corresponding to different chain conformations [36]. P(VDF-TrFE) generally exhibits good piezo- and ferro-electric behavior and a single all-trans polar crystalline phase that is stable at room temperature. The crystal structure of P(VDF-TrFE) co-polymer nanofibers was characterized by using XRD, as shown in Figure 1d, the characteristic peak locates at 2*θ* = 19.8°, which comes from the sum of the (110) and (200) diffraction [36]. The polymeric crystallinity of the nanofibers was further verified by Fourier Transform Infrared spectrum (FTIR) in the wavenumber range of 750–1500 cm^−1^, shown in Figure 1e. The transmission peaks are found at 510, 840, 1279 cm^−1^, which illustrates associated intense bands and all the peaks belong to the polar β-phase PVDF. By contrast, the bands of the non-polar α-phase (532, 612, 796, 854, 870 cm^−1^) are not appreciable, and the peaks in 766 cm^−1^ and 976 cm^−1^ are very weak. according to Gregorio et al. [37], the relative fraction of the β-phase in a sample containing just α and β-PVDF is:

(1) $$F\left(\beta \right)=\frac{A_{\beta }}{\left(K_{\beta }/K_{\alpha }\right)A_{\alpha }+A_{\beta }}$$

where *F(β)*, represents the β-phase content; *A_α_* and *A_β_* are the absorbance at 766 and 840 cm^−1^, *K_α_* and *K_β_*, at the respective wavenumber of 766 and 840 cm^−1^. which values are 6.1 × 10^4^ and 7.7 × 10^4^ cm^2^·mol^−1^, respectively. In this way, it is assumed that FTIR absorption follows the Lambert-Beer law and calculated the absorption coefficients, *A_α_* value is 0.05 and *A_β_* value is 0.20, respectively. Hence, the percentage of the β-phase in the sample is about 76%

**Figure 2:**
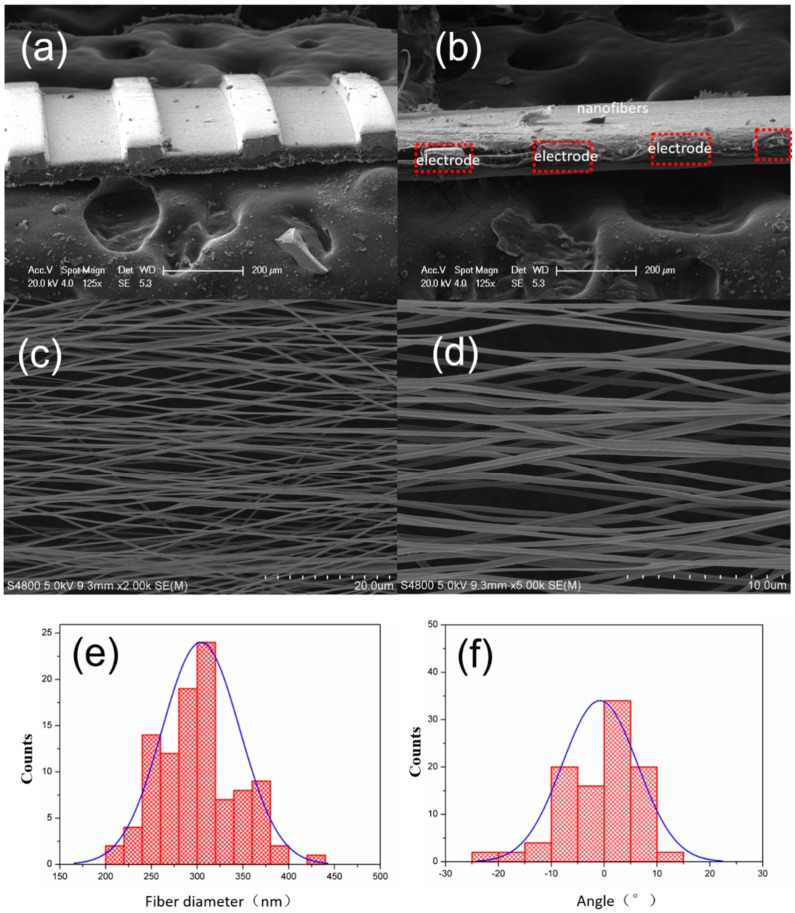
(**a**) SEM image of the flexible parallel copper electrode, (**b**) side view SEM image of the P(VDF-TrFE) nanofibers and electrode after electrospinning process, (**c**) top view SEM image of the nanofibers, (**d**) enlarged SEM image of the nanofibers, (**e**) typical fiber diameter distribution statistics, (**f**) fiber orientation distribution statistics.

Figure 2a presents scanning electron microscopy (SEM) images of the flexible parallel copper electrode, it clearly shows that the electrodes have uniform size. And the width of the electrode is about 100 μm, the gap of the electrode is about 200 μm. The height of the electrode is about 50 μm. Figure 2b shows the side view SEM image of P(VDF-TrFE) nanofibers and electrode after the electrospinning process. The fiber is fully stretched across the gap between two electrodes, and the thickness of the fiber is less than the electrode height. Figure 2c,d show the top view SEM images of the nanofibers, the diameter of the P(VDF-TrFE) nanofibers is uniform, the fibers are laterally aligned on the parallel electrodes. We used Image-pro plus software to calculate the diameter and orientation distribution, as shown in Figure 2e,f, it can be seen that electrospinning nanofibers diameter distribution range is 200–450 nm, concentrating between 250–350 nm, while angle distribution range is −15°~20°, mainly concentrating in the area of ±10°. 

**Figure 3:**
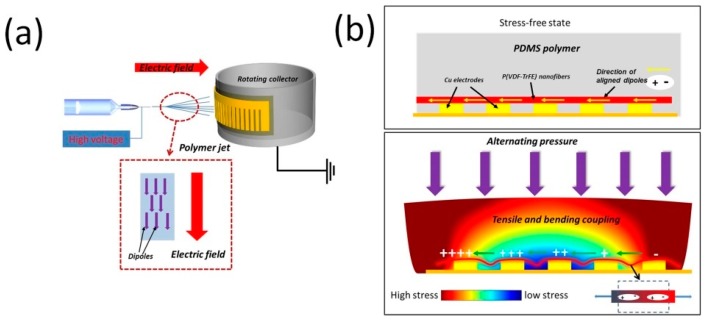
(**a**) Schematic of the electrospinning experimental setup. (**b**) Cross-sectional view of the generator.

The piezoelectric nanogenerator and power generation mechanism are illustrated in Figure 3a,b. First, the nanofibers are prepared by electrospinning, which provides a high electric field for the preferential orientation of molecular dipoles (CH_2_/CF_2_-dipoles) in PVDF and its copolymer, then the PENG is formed by establishing electrical contacts to two sides of the parallel copper electrode and packed with elastic polydimethylsiloxane (PDMS) polymer. After that, dynamic pressure is applied on the top surface of the PENG, which causes deformations of P(VDF-TrFE) nanofibers through the PDMS matrix. Then surface electric charges are generated due to the piezoelectric effect. Two models (*d_31_* and *d_33_*) are widely used for piezoelectric devices. In this case, because the direction of the generated electric signals is parallel to the applied stress/strain direction, model *d_33_* is adopted. Therefore, the induced open-circuit voltage can be expressed as follows [38]:

(2) $$V_{3j}=\frac{\sigma_{j}d_{3j}L_{j}}{\varepsilon_{0}\varepsilon^{T}}\left[V\right]$$

where *d_33_* is the piezoelectric charge constant, *σ_j_* is the mechanical stress, and *L* is the distance between two electrodes, *ε_0_* is the permittivity of vacuum, *ε* is the relative dielectric constant. 

A voltage difference between the two adjacent electrodes was induced due to the separation of charge. The nanofibers between each pair of adjacent electrodes worked as a unit cell, if the piezoelectric potential *V* is high enough, the electrons will flow from one electrode to another adjacent electrode to balance the electric field induced by dipoles. On the contrary, if the stress is released, the P(VDF-TrFE) will return from a flexed position to its original state and the piezoelectric potential between the two sides disappears. Simultaneously, the accumulated charges will flow back in the opposite direction, generating a reversed electrical signal. The repeat taping on the device generates output-voltages pulses. In a parallel electrodes situation, those cells were connected in series, which could enhance the performance of the device. 

**Figure 4:**
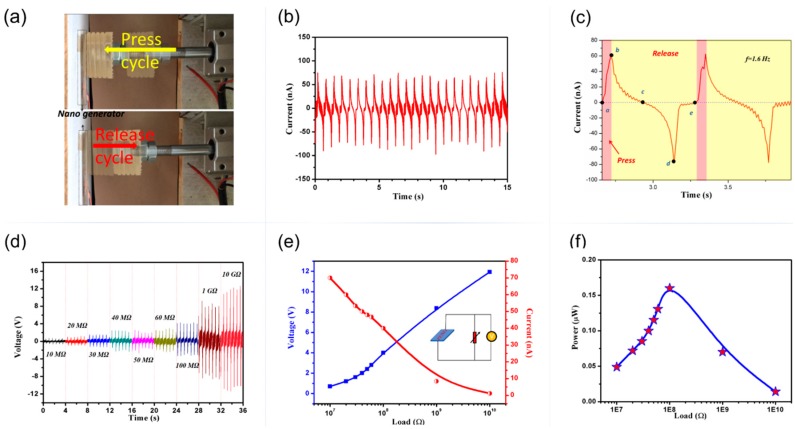
(**a**) Scheme of the press cycle and release cycle of mechanical loading equipment, (**b**) the short-circuit current of PENG during pressing-releasing cycles, (**c**) signal analysis corresponding to the cyclic pushing–releasing process, (**d**) output voltages from PENG with different load resistances, (**e**) the variation of output voltages and calculated currents across load resistances (inset is the corresponding circuit), (**f**) power density across different load resistances.

The piezoelectric performance of the device was tested on a purpose-built platform. A controlled dynamic impact was programmed to apply mechanical force by periodically pressing and releasing the device, the periodic pressure applied on PENG is about 2 kPa, as shown in Figure 4a. The induced output voltage was collected using a digital storage oscilloscope (TDS 2012B, Tektronix, Cleveland, OH, USA) and the short circuit current was measured by an electrochemical workstation (CHI 660 C, CH instruments, Shanghai, China). The resulting short circuit current and output voltage, detected at a loading frequency *f* = 1.6 Hz are shown in Figure 4b,f, respectively. The peak-peak short-circuit current is about 150 nA. The current generation mechanism is shown in Figure 4c: During the press cycle (part a–b), the nanofibers deformed from the original state (point a) to the maximum state (point b). The deformation quantity of the nanofibers was controlled and affected by external force. It increased from point a to b when pushing ahead, and reached highest in point b when the compression quantity was biggest. During the release cycle (part b–e), the stress released, the P(VDF-TrFE) nanofibers returned from the maximum strained position (point b) to the original strain-free state (point e), and the piezoelectric potential between the two sides disappeared. Simultaneously, the accumulated charges flowed back in the opposite direction, generating a negative electrical signal. The time of the pushing between two pulses peak was about 0.32 s, which was corresponding to the loading frequency of the external force. 

The variation of output voltages performance and calculated current signals from PENG across different load resistances are illustrated in Figure 4d,e. The results are obtained by measured the instantaneous output voltages of the PENG with external load resistance R from 10 MΩ–10 GΩ under a pressure of ~2 kPa at 1.6 Hz. The output voltages V increased with increasing the load resistance and reached a saturate value about 12 V at the load resistance of 10 GΩ (blue line). Whereas the current decreased with the increasing load resistances (red line). As a power source, our generator can achieve a maximum output power about 0.16 μW at the resistance of 100 MΩ.

**Figure 5:**
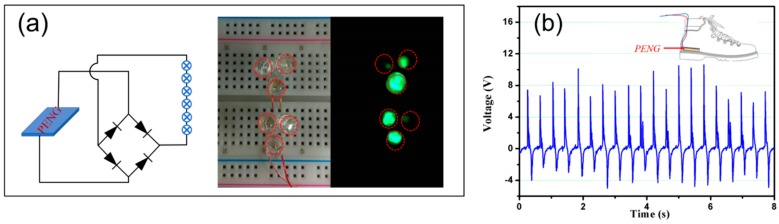
(**a**) The lighted LEDs and associated circuit diagram: (**b**) Output voltage generated by human walking.

Mechanical energy exists widely in the surrounding environment, most of which is wasted in daily life. As a wearable device application, here, we tried to apply a periodic dynamic load on the top of the PENG with human fingers, and the power can light up 6 LEDs immediately as illustrated in Figure 5a. A dynamic demonstration is shown in the video (Supporting Information Video S1). As we know, human walking offers an additional mechanical energy source for step counting or wearable device charging. We tried to place PENG inside a shoe, as shown in Figure 5b, the generated maximum voltage output is about 8 V during a 75 kg human jumping at a speed of 2~3 step/s. These results indicate that the PENG has the potential to be used as a mechanical force sensor for human movement monitor, such as step counting, etc., or an inexhaustible power source for self-powered wearable devices.

## 4. Conclusions

In summary, the aligned and high-density piezoelectric P(VDF-TrFE) nanofibers were prepared by electrospinning methods with parallel electrodes, which can be fabricated into a flexible piezoelectric nanogenerator easily. Under an applied pressure, the PENG can generate a maximum output voltage as high as 12 V at high load resistance and the peak-peak short-circuit current over 150 nA, which are significantly higher than other reported PENGs. The PENG can light up 6 LEDs and harvest the energy from human walking. This PENG provides great potential of the practical application for wearable electronic equipment.

## Supplementary Materials

The following are available online at https://www.mdpi.com/2072-666X/10/5/302/s1, Video S1: The PENG can light 6 LEDs.

## References

[1] Zhao Y., Liao Q., Zhang G., Zhang Z., Liang Q., Liao X., Zhang Y. (2015). High output piezoelectric nanocomposite generators composed of oriented BaTiO_3_ NPs@PVDF. Nano Energy.

[2] Huang T., Wang C., Yu H., Wang H., Zhang Q., Zhu M. (2015). Human walking-driven wearable all-fiber triboelectric nanogenerator containing electrospun polyvinylidene fluoride piezoelectric nanofibers. Nano Energy.

[3] Nguyen V., Zhu R., Yang R. (2015). Environmental effects on nanogenerators. Nano Energy.

[4] Dagdeviren C., Joe P., Tuzman O.L., Park K., Lee K.J., Shi Y., Huang Y., Rogers J.A. (2016). Recent progress in flexible and stretchable piezoelectric devices for mechanical energy harvesting, sensing and actuation. Extrem. Mechanics Lett..

[5] Dagdeviren C., Li Z., Wang Z.L. (2017). Energy harvesting from the animal/human body for self-powered electronics. Annu. Rev. Biomed. Eng..

[6] Ponmozhi J., Frias C., Marques T., Frazao O. (2012). Smart sensors/actuators for biomedical applications: Review. Measurement.

[7] Wang X., Yang B., Liu J., Zhu Y., Yang C., He Q. (2016). A flexible triboelectric-piezoelectric hybrid nanogenerator based on P(VDF-TrFE) nanofbers and PDMS/MWCNT for wearable devices. Sci Rep..

[8] Jung W.S., Lee M.J., Kang M.G., Moon H.G., Yoon S.J., Baek S.H., Kang C.Y. (2015). Powerful curved piezoelectric generator for wearable applications. Nano Energy.

[9] Shirinov A.V., Schomburg W.K. (2008). Pressure sensor from a pvdf film. Sens. Actuators A Phys..

[10] Dagdeviren C., Yang B.D., Su Y., Tran P.L., Joe P., Anderson E.K., Xia J., Doraiswamy V., Dehdashti B., Feng X. (2014). Conformal piezoelectric energy harvesting and storage from motions of the heart, lung, and diaphragm. Proc. Natl. Acad. Sci. USA.

[11] Lin L., Xie Y., Niu S., Wang S., Yang P.K., Wang Z.L. (2015). Robust triboelectric nanogenerator based on rolling electrification and electrostatic induction at an instantaneous energy conversion efficiency of ~ 55%. Acs Nano.

[12] Wu Y., Xue W., Yang Y., Zhong L.W. (2015). Hybrid energy cell for harvesting mechanical energy from one motion using two approaches. Nano Energy.

[13] Wang S., Lin L., Xie Y., Jing Q., Niu S., Wang Z.L. (2013). Sliding-triboelectric nanogenerators based on in-planecharge-separation mechanism. Nano Lett..

[14] Siddiqui S., Kim D.-I., Duy L.T., Nguyen M.T., Muhammad S., Yoon W.-S., Lee N.-E. (2015). High-performance flexible lead-free nanocomposite piezoelectric nanogenerator for biomechanical energy harvesting and storage. Nano Energy.

[15] Chang J., Dommer M., Chang C., Lin L. (2012). Piezoelectric nanofibers for energy scavenging applications. Nano Energy.

[16] Gheibi A., Latifi M., Merati A.A., Bagherzadeh R. (2014). Piezoelectric electrospun nanofibrous materials for self-powering wearable electronic textiles applications. J. Polym. Res..

[17] Song S., Yun K.S. (2015). Design and characterization of scalable woven piezoelectric energy harvester for wearable applications. Smart Mater. Struct..

[18] Elahi H., Eugeni M., Gaudenzi P. (2018). A review on mechanisms for piezoelectric-based energy harvesters. Energies.

[19] Wang Z.L., Song J.H. (2006). Piezoelectric nanogenerators based on zinc oxide nanowire arrays. Science.

[20] Hansen B.J., Liu Y., Yang R., Wang Z.L. (2010). Hybrid nanogenerator for concurrently harvesting biomechanical and biochemical energy. ACS Nano.

[21] Han M., Zhang X.S., Meng B., Liu W., Tang W., Sun X., Wang W., Zhang H. (2013). R-shaped hybrid nanogenerator with enhanced piezoelectricity. ACS Nano.

[22] Wu W., Bai S., Yuan M., Qin Y., Wang Z.L., Jing T. (2012). Lead zirconate titanate nanowire textile nanogenerator for wearable energy-harvesting and self-powered devices. ACS Nano.

[23] Pi Z., Zhang J., Wen C., Zhang Z.B., Wu D. (2014). Flexible piezoelectric nanogenerator made of poly(vinylidenefluoride-co-trifluoroethylene) (PVDF-TrFE) thin film. Nano Energy.

[24] Jiang Y., Gong L., Hu X., Zhao Y., Chen H., Feng L., Zhang D. (2018). Aligned P(VDF-TrFE) Nanofibers for Enhanced Piezoelectric Directional Strain Sensing. Polymers.

[25] Park S., Kim Y., Jung H., Park J.-Y., Lee N., Seo Y. (2017). Energy harvesting efciency of piezoelectric polymer flm with graphene and metal electrodes. Sci Rep..

[26] Orrego S., Shoele K., Ruas A., Doran K., Caggiano B., Mittal R., Kang S.H. (2017). Harvesting ambient wind energy with an inverted piezoelectric flag. Appl. Energy.

[27] Fashandi H., Abolhasani M.M., Sandoghdar P., Zohdi N., Li Q., Naebe M. (2016). Morphological changes towards enhancing piezoelectric properties of PVDF electrical generators using cellulose nanocrystals. Cellulose.

[28] You S., Shi H., Wu J., Shan L., Guo S., Dong S. (2016). A flexible, wave-shaped P(VDF-TrFE)/metglas piezoelectric composite for wearable applications. J. Appl. Phys..

[29] Kim S., Towfeeq I., Dong Y., Gorman S., Rao A.M., Koley G. (2018). P(VDF-TrFE) Film on PDMS Substrate for Energy Harvesting Applications. Appl. Sci..

[30] Shi X., Zhou W., Ma D., Ma Q., Bridges D., Ma Y., Hu A. (2015). Electrospinning of nanofibers and their applications for energy devices. J. Nanomater..

[31] You S., Ai L., Li D., Huang H., Chen W.P., Liu W., Guo S., Zhao X.Z. (2013). Enhanced electrical properties of composite nanostructures using BiFeO_3_ nanotubes and ferroelectric copolymers. Mater. Lett..

[32] Sun C., Shi J., Bayerl D.J., Wang X. (2011). PVDF microbelts for harvesting energy from respiration. Energy Environ. Sci..

[33] Fang J., Wang X., Lin T. (2011). Electrical power generator from randomly oriented electrospun poly (vinylidene fluoride) nanofibre membranes. J. Mater. Chem..

[34] Persano L., Dagdeviren C., Su Y., Zhang Y., Girardo S., Pisignano D., Huang Y., Rogers J.A. (2013). High performance piezoelectric devices based on aligned arrays of nanofibers of poly(vinylidenefluoride-co-trifluoroethylene). Nat. Commun..

[35] Li D., Wang Y., Xia Y. (2010). Electrospinning nanofibers as uniaxially aligned arrays and layer-by-layer stacked films. Adv. Mater..

[36] Martins P., Lopes A.C., Lanceros-Mendez S. (2014). Electroactive phases of poly(vinylidene fluoride): Determination, processing and applications. Prog. Polym. Sci..

[37] Gregoriojr R., Ueno E.M. (1999). Effect of crystalline phase, orientation and temperature on the dielectric properties of poly (vinylidene fluoride) (PVDF). J. Mater. Sci..

[38] Chen X., Xu S., Yao N., Xu W., Shi Y. (2009). Potential measurement from a single lead zirconate titanate nanofiber using a nanomanipulator. Appl. Phys. Lett..

